# Changes to the Gut Microbiome in Young Children Showing Early Behavioral Signs of Autism

**DOI:** 10.3389/fmicb.2022.905901

**Published:** 2022-07-28

**Authors:** Jacquelyn Jones, Stacey N. Reinke, Mahsa Mousavi-Derazmahalleh, Debra J. Palmer, Claus T. Christophersen

**Affiliations:** ^1^Trace and Environmental DNA Laboratory, School of Molecular and Life Sciences, Curtin University, Bentley, WA, Australia; ^2^The Western Australian Human Microbiome Collaboration Centre, Curtin University, Bentley, WA, Australia; ^3^Centre for Integrative Metabolomics and Computational Biology, School of Science, Edith Cowan University, Joondalup, WA, Australia; ^4^Telethon Kids Institute, University of Western Australia, Nedlands, WA, Australia; ^5^School of Medicine, University of Western Australia, Crawley, WA, Australia; ^6^School of Medical and Health Sciences, Edith Cowan University, Joondalup, WA, Australia

**Keywords:** microbiome, gut-brain-microbiota axis, autism spectrum disorder, short-chain fatty acid, stool form

## Abstract

The human gut microbiome has increasingly been associated with autism spectrum disorder (ASD), which is a neurological developmental disorder, characterized by impairments to social interaction. The ability of the gut microbiota to signal across the gut-brain-microbiota axis with metabolites, including short-chain fatty acids, impacts brain health and has been identified to play a role in the gastrointestinal and developmental symptoms affecting autistic children. The fecal microbiome of older children with ASD has repeatedly shown particular shifts in the bacterial and fungal microbial community, which are significantly different from age-matched neurotypical controls, but it is still unclear whether these characteristic shifts are detectable before diagnosis. Early microbial colonization patterns can have long-lasting effects on human health, and pre-emptive intervention may be an important mediator to more severe autism. In this study, we characterized both the microbiome and short-chain fatty acid concentrations of fecal samples from young children between 21 and 40 months who were showing early behavioral signs of ASD. The fungal richness and acetic acid concentrations were observed to be higher with increasing autism severity, and the abundance of several bacterial taxa also changed due to the severity of ASD. Bacterial diversity and SCFA concentrations were also associated with stool form, and some bacterial families were found with differential abundance according to stool firmness. An exploratory analysis of the microbiome associated with pre-emptive treatment also showed significant differences at multiple taxonomic levels. These differences may impact the microbial signaling across the gut-brain-microbiota axis and the neurological development of the children.

## Introduction

Autism spectrum disorder (ASD) is a complex, chronic, neurological developmental disorder that is characterized by impairments to social interaction, as well as repetitive stereotyped behaviors (Fakhoury, 2015). Among children and adolescents, this disorder affects ~1 in every 58 to 166 children and is 4 times more prevalent in males than females (Iglesias–Vázquez et al., 2020). Autism and its severity are diagnosed by assessing the core behavioral symptoms using several different diagnostic tools, which have been reviewed elsewhere (Falkmer et al., 2013). Children with ASD show high comorbidity of gastrointestinal (GI) disturbance, which may be linked with neurological symptom severity (Chaidez et al., 2015), and infants who would be diagnosed with ASD are also more likely to have experienced GI symptoms, including constipation and food intolerance between 6 – 18 months of age (Bresnahan et al., 2015). Children with autism are also often self-restricting when it comes to dietary preferences (Hyman et al., 2020); and diet is known to be an important factor in driving the composition of the microbiome in both autistic (Yap et al., 2021) and healthy individuals (Xu and Knight, 2015; Heiman and Greenway, 2016; Beaumont et al., 2017).

The gut microbiome is a complex and critical community (Dave et al., 2012), which has been well-described (in terms of the bacterial residents) for healthy children and adults (Gilbert et al., 2018), and significant differences in the composition of the fecal microbiome between children with autism and neurotypical children have also been shown (Kang et al., 2013; de Angelis et al., 2015). It is also now widely recognized that early-life colonization patterns can have long-lasting effects on human health (Mesa et al., 2020) and that microbes play a crucial role in maintaining the normal functioning of their host (Gilbert et al., 2018). This is achieved in part by the production of exclusive microbial metabolites, including short-chain fatty acids (SCFA), which, like the microbial members, are found in dynamic quantities in the gut. The gut ecosystem will fluctuate in response to diet and nutrient availability, the presence of antimicrobials, which affects patterns of cross-feeding (Ríos-Covián et al., 2016), as well as physical activity, and hygiene practices (Levy et al., 2017). Lifestyle choices can, therefore, result in shifts or impairments to the composition of the microbial community, and deficits in digestion, absorption, or metabolic imbalances can also feed into GI disturbance and exacerbate a breakdown of the microbial community. Under such conditions, which favor the growth of opportunistic pathogenic bacteria, certain microbial products in the gut can induce an enhanced pro-inflammatory response, which can compromise the integrity of the gut epithelial barrier (Martin et al., 2018).

Some bacteria-derived toxins can enter the bloodstream through leaky gastrointestinal barriers and then pass through the blood-brain barrier (BBB). These toxins include enterotoxins and lipopolysaccharides (Lukiw, 2020), and phenols, such as 3-hydroxyphenyl acetic acid (Velásquez-Jiménez et al., 2021), whereas SCFA plays a part in regulating the BBB (Parker et al., 2020). These events, which begin in the gut, can have far-reaching effects due to bidirectional communication, which takes place within the gut-brain-microbiota axis and involves signaling in neural, endocrine, and immune systems (Martin et al., 2018). The microbiome can be affected by modifications to gut motility, permeability, and intestinal secretions, which are directed by the brain (Martin et al., 2018), and there is evidence for the microbiome to play a role in both the development and long-term functioning of the brain (Sharon et al., 2016). Due to the potential disruption of normal brain development *via* the gut-brain-microbiota axis (de Angelis et al., 2015), it has been proposed that the gut microbiota plays a role in both the GI- and developmental symptoms that affect autistic children (de Angelis et al., 2015; Cryan et al., 2020).

An exciting prospect of the causal role the microbiome may play in the development and severity of autism is that the microbiome is modifiable (Halmos et al., 2015; Pham et al., 2021). A temporary reduction in symptom severity has been observed after modulation of the gut microbiome using either antibiotic (Sandler et al., 2000) or fecal microbiome transplantation (Kang et al., 2017). Probiotics have also been shown to improve stool consistency and behavioral scores of autistic children (Parracho et al., 2010). If autism can be detected earlier, behavioral and dietary interventions can be implemented earlier, and would potentially be more effective. While the fecal microbiome of adolescent and older children with ASD has repeatedly shown shifts to the bacterial and fungal microbial community, it is still unclear whether these characteristic shifts might be detectable earlier. In a recent systematic review on the gut microbiota of children with autism, only 4 of 18 investigations included children at 2 years of age (Iglesias–Vázquez et al., 2020). In this study, we analyzed stool samples from young children who took part in a larger study that compared two pre-emptive intervention treatments (Whitehouse et al., 2021). The present work aimed to characterize the gut microbiome and SCFA concentrations in young children who were showing behaviors in the first year of life that are associated with a later autism diagnosis (ASD-risk behaviors). The relationship between the microbiome composition and function and its association with clinical measures for autism and neurodevelopment was investigated. We have also explored the microbiome for any association with the pre-emptive intervention that these young children were receiving, which may inform larger studies in the future.

## Methods

### Study Design and Sample Collection

Samples were collected from young children who were enrolled in a broader study (Whitehouse et al., 2021), which was approved by the Child and Adolescent Health Service (HREC Ref: 2016008EP) in Perth, Western Australia. This mentioned study recruited infants between 9 and 14 months who were showing early social-communication delays as determined by Social Attention and Communication Surveillance–Revised (SACS-R) 12-month checklist. In this randomized control trial (RCT), the infants were randomized to one of two intervention arms: 1) iBASIS-VIPP – a parent-mediated video-aided intervention supporting parent-child interaction, or 2) usual community care (UCC) – which was varied and was comprised of services recommended by local health professionals, or included those with no additional treatment (Whitehouse et al., 2021). As part of this larger study, autism symptom severity and general development of the children was assessed at the time of stool sample collection using both the Mullens Scale of Early Learning (MSEL) (Mullen, 1995) and the Autism Diagnostic Observation Schedule, second edition (ADOS-2) (Lord et al., 2012) diagnostic tools (Table 1). ADOS-2 total scores were converted to calibrated severity scores (CSS), which range from 1 to 10 points (Shumway et al., 2012). The CSS scale were from scores 6–10 moderate-to-severe concern/autism classification, 4–5 mild-to-moderate concern/autism spectrum classification, and 1–3 little-to-no concern/non-spectrum classification.

**Table 1:** Enrolment, follow-up time points, and behavioral testing that took place in the AICES RCT.

**AICES study timepoints**	**Enrollment**	**Follow up 1**	**Follow up 2**	**Follow up 3**
Age (months)	9–14	15–20	21–28	33–38
ADOS-2 scoring	x	x	x	
MSEL scoring	x	x	x	x
**Current study timepoints**			**Timepoint A**	**Timepoint B**
number of single stool samples received	3	15		
number of replicate stool samples received	6	6		
Boys: Girls			8:1	15:6

*The number of stool samples received from this RTC and used in this current fecal microbiome study is also indicated. Six children provided stool samples at both timepoints A and B*.

Stool samples were collected as part of the formerly mentioned study in Perth. Parents were instructed to collect a stool sample from a nappy or from a plastic lining that covered the toilet, and, preferably, free of urine, in a sterile screw-top container. The stool samples were placed inside a sealable bag and frozen immediately in the household freezer. A provided freezer bag was used to transport the sample to the clinical assessment site (CliniKids, SUBIACO/Perth Children's Hospital, NEDLANDS) where they were frozen at −80°C until transferred to Curtin University on dry ice. A total of 30 stool samples were collected from 24 children at two time points during the RCT. Nine samples were collected 1 year after the study baseline when the children were between 21 and 28 months of age, and 21 samples were collected 2 years post-baseline when children were between 33 and 40 months of age. Six of the 24 children provided stool samples at both one- and two-years post-baseline (Table 1). Those stool samples were used in this current fecal microbiome study, for which approval was granted by Human Research Ethics Committee (approval number HRE2020-0127) from Curtin University, Western Australia, and all research was conducted in accordance with the relevant regulations and guidelines.

### Fecal DNA Extraction and Short-Chain Fatty Acid Quantification

Fecal material was thawed at 4°C and homogenized manually for 1 min before sample collection for SCFA quantification (1 g ±0.1), and microbial sequencing (0.25 g ±0.05). The Bristol Stool Form Scale (BSFS) was used to categorize each stool form during homogenization (Mínguez Pérez and Benages Martínez, 2009). DNA was extracted from fecal samples immediately after homogenization using the QIAamp PowerFecal Pro DNA kit (QIAGEN, Hilden, Germany) using the IRT protocol for QIAcube (QIAGEN), according to the manufacturer's instructions with three modifications: (1) before adding stool sample, three 3.5-mm glass beads (Biospec) were added to bead-beating tubes, (2) after step 1, tubes were vortexed for ~20 s to incorporate beads and stool, and (3) followed by heating at 65°C for 10 min. Extraction controls were also processed following the same protocol as frozen stool samples. Fecal samples for SCFA analysis were frozen at −80°C immediately after homogenization, and then transferred on dry ice to the Science Analytical Facility at Edith Cowan University, Western Australia for SCFA quantification as previously described (Jones et al., 2021).

### Bacterial and Fungal Library Preparation and Sequencing

Bacterial DNA and gut microbiome mock community (https://www.atcc.org/products/msa-1006) were amplified using 16S primers 515F (Turner et al., 1999) and 806R (Caporaso et al., 2011), while fungal DNA was amplified using ITS2 primers FSeq and RSeq (Heisel et al., 2015), each with a 6–8 bp unique barcode. The PCR reactions, library preparation, and sequencing were performed according to methods previously described (Jones et al., 2021).

### Deconvolution and Data Quality Filtering

Sequences were demultiplex using unique molecular barcodes with no mismatches allowed before they were removed along with primer sequences using Cutadapt (Martin, 2011). Quality filtering using DADA2 (Callahan et al., 2016) was performed as previously described (Jones et al., 2021). The Genome Taxonomy reference database (Version 202) was formatted for use with DADA2 (https://zenodo.org/record/4735821#.YN18Om4RWis), and the UNITE general FASTA release for fungi Version 8.3 (Kõljalg et al., 2005) were used to classify 16S and ITS2 sequence variants, respectively, each with a minimum of 80% bootstrap. Species were then assigned to 16S sequences with 100% identity using the same reference database. The bacterial and fungal species assignments for the top 50 ASVs were confirmed by BLAST using the same two databases, at 100% identity. ASVs with up to three matches were annotated to include all three potential species assignments, whereas any ASV with more than three identical matches was annotated with the genus name followed by “spp.” Identical matches to species' ID numbers were not included. If taxa are not fully resolved to a lower rank, the lowest available rank name and sp., gen., or fam. have been annotated for each lower taxonomic level. The package microDecon (McKnight et al., 2019) was used to remove contamination from all sample sequences with one run of the function remove.count. Lastly, any ASVs with unassigned phylum, or with low prevalence (1 read in 5% of samples for bacterial ASVs and 1 read in 2.5% of fungal sample ASVs) were filtered out, as were fungal samples (1 sample) with <1,000 reads. Performing decontamination in conjunction with filtering has been recommended (Cao et al., 2021), and, therefore, these non-aggressive filtering and decontamination thresholds were chosen to balance noise reduction associated with sparse reads while retaining less abundant but potentially important species.

### Statistical Analysis

To characterize the difference between autistic and non-spectrum children using CSS, the children were placed into the category defined by their scores: CSS from 6 to 10 ASD, CSS from 4 to 5 non-autism autism spectrum disorder (NAASD), and CSS from 1 to 3 no developmental concern (NDC). Also, because stool form has previously been shown to be associated with alpha diversity and SCFA concentrations, the samples were placed into three groups determined by the Bristol stool form scale. Stool scoring of 1–2 was considered firm, 3–4 was considered normal, and 5–6 was considered loose. No stools were scored as 7 in this study. Beta diversity was used to assess differences between CSS and intervention groups, and stool form using Euclidian distances of center log-ratio transformed counts and visualized using Principal coordinates analysis (PCoA). PERMANOVA was performed in PRIMER-e v7 (Anderson et al., 2008) with 9,999 unrestricted permutations of the raw data and type 3 sum of squares. ANOVA was used to determine differences in SCFA concentration between CSS groups using the Tukey's multiple comparison method to adjust *p* values. For all SCFA concentrations, Q-Q plots were used to assume normality, and Levene's test was used to check for homogeneity of variance. The total SCFA concentration was summed from all acids, with the concentration of valeric acid set to 0 for 3 individuals, where the concentration was below detection.

Differential abundance testing between CSS groups at the ASV level was performed in DESeq2 (Love et al., 2014). At the family, genus, and species level, differences observed between the intervention group and CSS, were determined using the Mann-Whitney U test and Bristol stool groups using the Kruskal-Wallis test (all comparisons included only samples from timepoint B). The higher-order comparisons were performed only on dominant reads, which were those with a sum greater than 200 reads across all bacterial samples (372 ASV), and greater than 25 reads across all fungal samples (136 ASVs). The false discovery rate (FDR) due to multiple testing was corrected with a Benjamini-Hochburg adjustment (BH) (Benjamini and Hochberg, 1995). The core bacteriome consisting of species with a 20% prevalence and a relative abundance of over 0.01% were also determined in MicrobiomeAnalyst (Dhariwal et al., 2017). Pearson correlation was calculated for the linear regression between MSEL and alpha diversity estimates for bacterial ASV counts, with normality assumed by the Shapiro-Wilk normality test. A single sample was removed from this correlation analysis as no MSEL score was recorded for that sample.

Predictive functional profiling was inferred from the total bacterial 16S rRNA gene sequence data using Tax4Fun (Wemheuer et al., 2020). Here, SILVA reference sequences were mapped to KEGG orthologs (KO), and the profile was then filtered to include only a core group (>0.01% across all samples) of metabolic pathways, with photosynthesis (ko00195) also removed. Differences in the core metabolic profile between CSS groups were visualized in Primer using PCoA of Bray-Curtis similarity and using PCA in STAMP (Parks et al., 2014). Significant differences between individual pathways were also assessed in STAMP using two-sided Welch's test and the Benjamini-Hochberg FDR for multiple testing.

## Results

### Description of the Total Bacterial and Fungal Datasets

The bacteriomes from all children in this study were dominated by Firmicutes (62%), Bacteroidota (25%), Actinobacteriota (10%), and the mycobiome was dominated by Saccharomyces (92%) and Kazachatania (4%). A description of the data quality, including read depth (Supplementary Table 1), replicate sampling, and positive control in the form of a bacterial mock community (Supplementary Figure 1), is summarized in the supplementary results section.

### Microbiome Composition Assessed by Stool Form and Intervention Group

To explore the data, principal coordinates analysis (PCoA) was used to visualize beta diversity between stool form, age, and dominant taxa from stool samples at both time points, while differences between intervention groups were assessed only from those samples taken at time point B. A significant difference in the beta diversity of intervention groups was determined by PERMANOVA at the level of species (*p* = 0.014) genus (*p* = 0.008), family (*p* = 0.006), and order (*p* = 0.008) (Supplementary Figure 2). A significant difference in the dispersion of Phyla around the centroid between the two intervention groups was also determined by PERMDISP (*p* = 0.04). The difference in community composition seemed to be driven by a higher abundance of Lachnospirales (*p* = 0.005, FDR = 0.12) in the iBASIS-VIPP intervention group. Moreover, 2 families, 8 genera, and 13 species were also differentially abundant between the two intervention groups (Supplementary Table 2).

**Figure 1:**
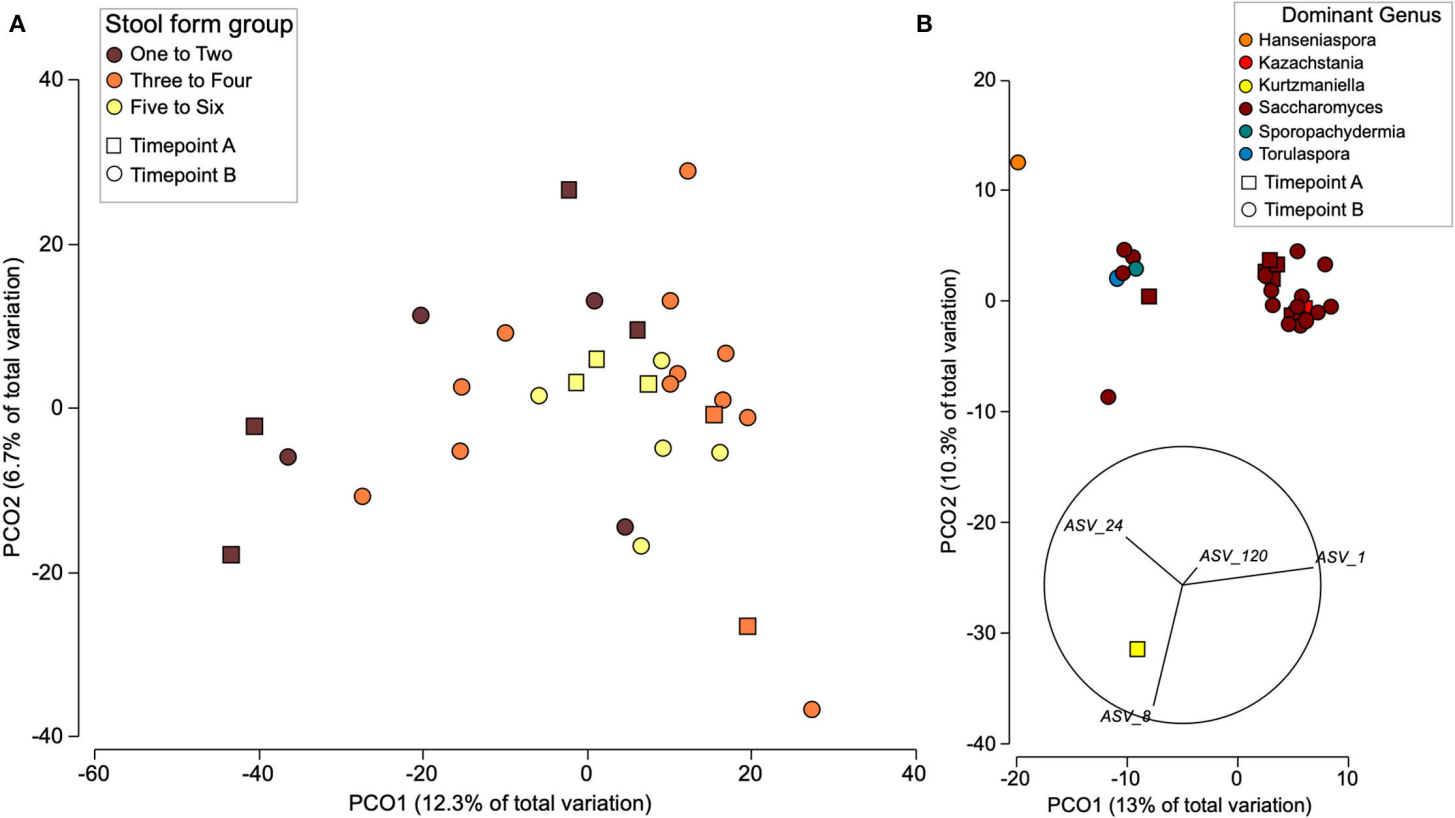
Beta diversity of bacterial **(A)** and fungal **(B)** communities from all individuals at both time points using PCoA. **(A)** Distribution of the bacterial communities is shown due to stool form and timepoint. **(B)** Fungal communities are displayed based on dominant taxa and timepoint with abundant ASVs plotted as vectors. Beta diversity was estimated from Euclidian distances between CLR transformed counts.

The age in months did not have a clear impact on sample beta diversity (Supplementary Figure 3), and, independent of timepoint, bacterial communities clustered according to stool form (Figure 1A), with firmer stool samples clustering separately from loose stool samples. Bacterial families with differential abundance according to stool form were detected (Supplementary Figure 4). Prior to FDR correction, the abundance of Butyricicoccaceae and Pasteurellaceae was significantly higher in loose stool, compared to firm stool (*p* > 0.05 FDR > 0.60); the abundance of Enterobacteriaceae was significantly higher in loose stool compared to normal stool (*p* = 0.03 FDR = 0.42); and the abundance of Monoglobales A UBA1381 was significantly lower in firm stool compared to normal or loose stool (*p* = 0.02, FDR = 0.42). A further six genera were identified to have lower abundance in firm stool compared to lose or normal stool (*p* < 0.05, FDR < 0.59), and 3 genera had lower abundance in normal stool compared to a firm or loose stool (*p* < 0.03, FDR0.44) (Supplementary Table 3). Fungal communities, on the other hand, did not group according to stool form but clustered according to the dominant ASVs (Figure 1B).

### Comparison of Microbiome Diversity Between Diagnosis Outcomes

**Figure 2:**
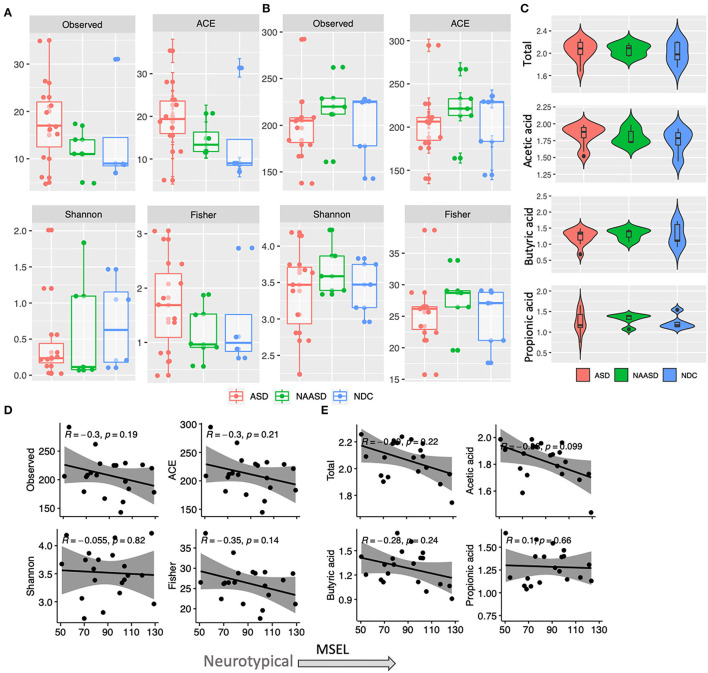
Differences in alpha diversity and SCFA concentration in young children according to autism severity. **(A)** Fungal community alpha diversity estimates according to CSS groups. **(B)** Bacterial community alpha diversity estimates according to CSS groups. **(C)** Total and individual SCFA concentration (log10) in CSS groups. **(D)** Association between MSEL score and bacterial alpha diversity based on Pearson correlation. **(E)** Association between MSEL score and SCFA concentration based on Pearson correlation.

The microbiome composition among CSS groups was compared at timepoint B only and showed that the proportions of bacterial phyla were similar among children with autism and children in the NDC group. However, the abundance of Actinobacteria in the NAASD group was considerably lower than in the other groups (Table 2). Strong interpersonal differences were observed between all children, and no significant differences were determined between CSS groups (*p* > 0.3). Similar alpha diversity was observed between CSS groups for both bacterial and fungal communities, and alpha diversity also showed no significant relationship with MSEL (Figure 2).

**Table 2:** Description of child and microbiome characteristics at time points A and B, and per diagnosis category.

**Time A (*n* = 9)**	**mean Age**	**Boys: Girls**	**Average stool form**	**Average MSEL**	**Act (%)**	**Bac (%)**	**Firm (%)**	**Pro (%)**
CSS								
ASD	25.5	3:1	2	74.8	2.9	29.2	66.9	0.5
NDC	24.2	5:0	3.8	108	2.5	30.3	64.5	0.9
**Time B (*****n*** **=** **21)**								
CSS								
ASD	36.4	8:3	3.1	78.7	15.3	17.5	65.6	0.3
NAASD	36.6	4:1	4.2	97.0	4.0	39.6	53.6	0.2
NDC*^a^	34.6	3:2	4.0	104.8	10.3	26.9	60.2	1.3
NDC*^b^	34.8	3:1	4.5	98.3				

A single sample was removed from the fungal data, but not the bacterial data, and, therefore, the group characteristics for NDC are different for bacterial ^*a^ and fungal ^*b^ datasets. Abbreviated phyla names are Act, Actinobacteriota; Bac, Bacteroidota; Firm, Firmicutes; Pro, Proteobacteria, while abbreviated CSS groups are ASD, Autism, NAASD, non-autistic autism spectrum disorder, NSD, developmental disorder.

Differential abundance testing was used to compare the microbiome composition at the ASV level among CSS groups at time point B only. In total, 19 bacterial and 4 fungal ASVs were detected with significantly different abundance after adjusting for multiple testing. These ASVs were identified by the lowest taxonomic rank available (Table 3), and most bacteria were classified as either Bacteroidia (Bacteroidales) or Clostridia, most of which were Lachnospiraceae, while most fungal taxa were classified as Saccharomycetales. Six bacterial ASVs were enriched in autistic children, half of which belonged to Lachnospiraceae, and ten bacterial ASVs were significantly enriched in the NDC group. Two different fungal ASVs were significantly enriched in both the mycobiome of NDC and children with autism. Differences in the abundance of genera and species between CSS groups were also determined. After correcting for multiple testing (FDR), there were no significant differences; however, before FDR, there were several taxa identified (Supplementary Table 4).

**Table 3:** Bacterial and fungal ASV's identified with DESeq2 as having significant changes in abundance between CSS groups.

**ASD compared to NDC group**			**NAASD compared to NDC group**		
**ASV# and lowest rank**		**log2 fold change**	**ASV# and lowest rank**		**log2 fold change**
Bacteroidaceae			Erysipelatoclostridiaceae		
287	Bacteroides	22.4	194	Erysipelatoclostridium sp	15.6
Burkholderiaceae			Debaryomycetaceae		
184	Parasutterella	6.7	14	Debaryomyces hansenii	28.0
Lachnospiraceae			Lachnospiraceae		
77	CHKCI001	5.3	127	Ruminococcus A faecicola	24.0
Bacteroidaceae			Lactobacillaceae		
172	Bacteroides finegoldii	−22.2	202	Lactobacillaceae sp	19.7
Christensenellales fam			Oscillospiraceae		
285	Christensenellales sp	−20.7	268	Oscillospiraceae sp	21.3
Lachnospiraceae			Veillonellaceae		
220	Blautia sp	−24.1	211	Veillonella parvula A	24.1
162	Blautia sp	−22.3	Bacteroidaceae		
58	Lachnospiraceae sp	−7.5	172	Bacteroides finegoldii	−24.6
150	Lachnospiraceae sp	−23.8	Lachnospiraceae		
Saccharomycetaceae			220	Blautia sp	−15.8
21	Saccharomyces sp	−22.1	162	Blautia sp	−17.6
13	Eremothecium sinecaudum	−20.3	58	Lachnospiraceae sp	−6.9
			150	Lachnospiraceae sp	−23.1
			Saccharomycetaceae		
			21	Saccharomyces sp	−19.6

*Positive fold changes are enriched in NDC, and negative fold changes are enriched in ASD or NAASD groups*.

### Temporal Changes in Fecal Samples From Six Individuals

**Figure 3:**
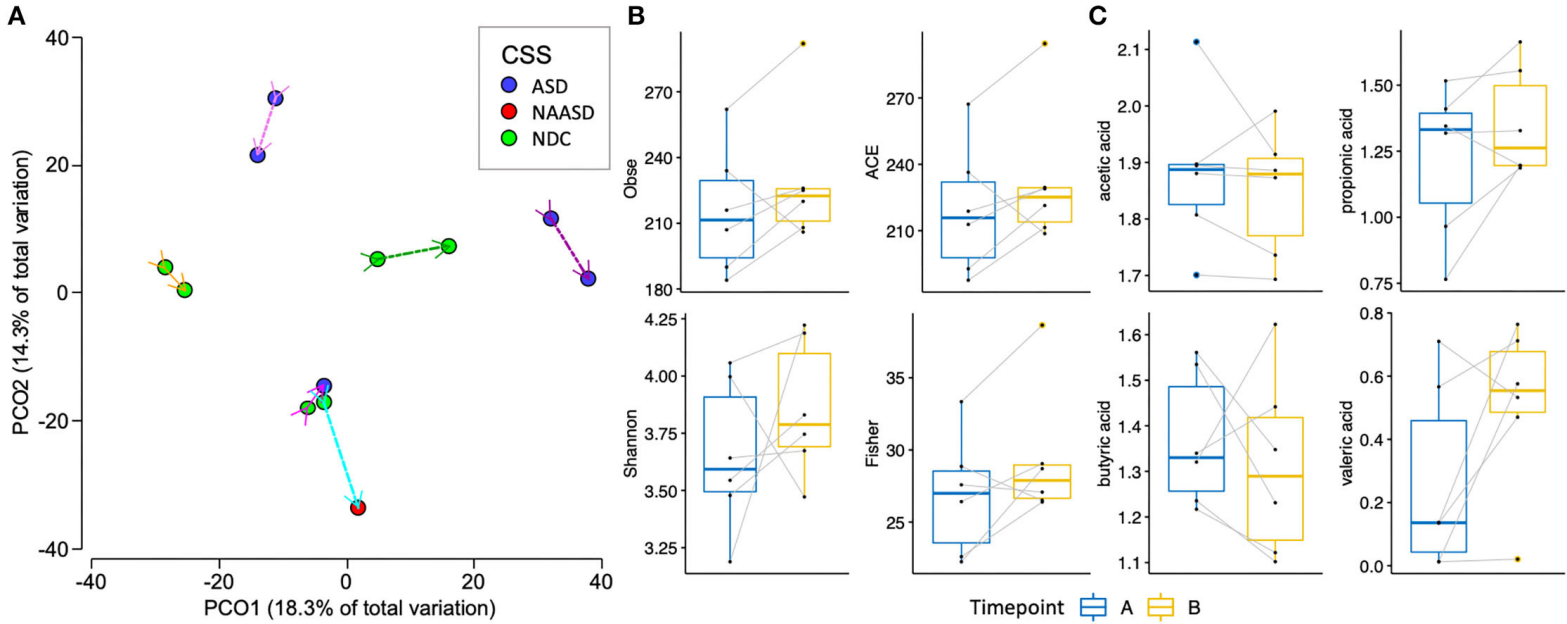
Changes in the gut microbiome of 6 children between an average of 24 months of age (timepoint A), and 36 months of age (timepoint B). **(A)** PCoA of bacterial beta-diversity based on Euclidian distances of CLR transformed counts and the trajectory of the microbiome across time is shown with a uniquely colored arrow for each individual. **(B)** Alpha diversity estimates between timepoint A and B. **(C)** SCFA concentrations between timepoint A and B.

Six children provided samples at both time point A and timepoint B. When the 12 samples were clustered using Euclidian distances (Figure 3), the samples were grouped based on participants, with a larger difference between one individual. This individual and one other additional individual each received a different ADOS-2 score based on their behavior at timepoint B than they had previously received at timepoint A. Overall the richness, diversity, and phylogenetic diversity were observed to be higher at the second time point when the children were on average 36 months of age. The SCFA concentrations did not shift in a particular direction between time points (Figure 3).

**Figure 4:**
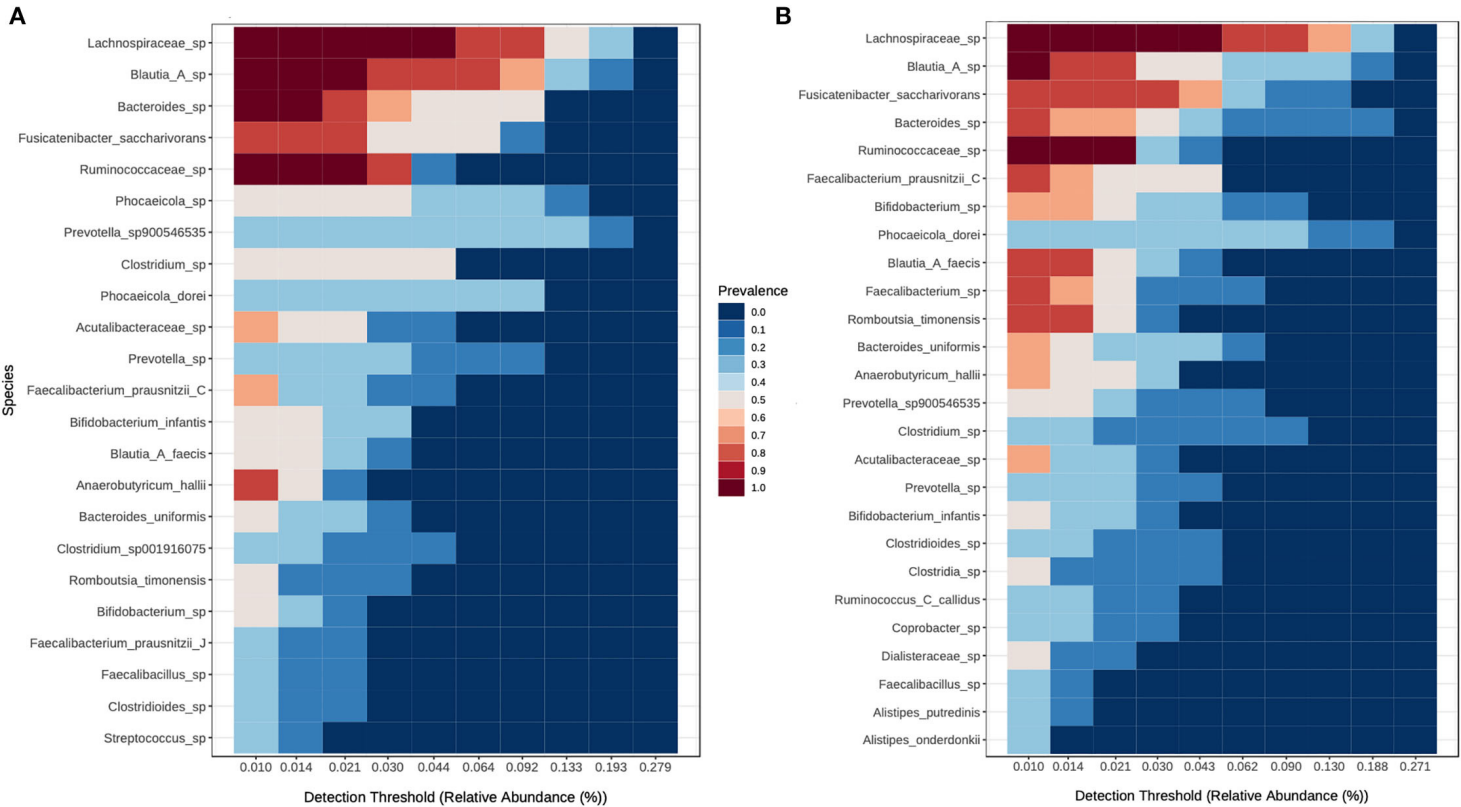
Shifts in the core microbiome of 6 children between timepoints. **(A)** Children between 21 and 28 months of age at time points A and **(B)** children between 33 and 40-months of age at time point B.

The common core bacteriome between all children at time points A and B was identified using prevalence and abundance filtering. A total of 46 species were identified as core members at time point A and 50 at time point B, with 39 of those species present in both core communities. *Blautia* A sp, Lachnospiraceae sp, and Ruminococcaceae sp remained highly prevalent between both time points, and 50 other species changed only marginally or not at all between timepoints. Eleven species that were present in the core microbiome at timepoint B were missing from the core at time point A, most of which were from Lachnospiraceae. *Phocaeicola* sp lost prevalence over time and *Blautia* A *faecis, Romboutsia timonensis/ilealis, Ruminococcus* C *callidus*, Clostridia sp, Dialisteraceae sp, and *Faecalibacterium* sp all increased in prevalence over time (Figure 4).

### SCFA Concentrations

**Figure 5:**
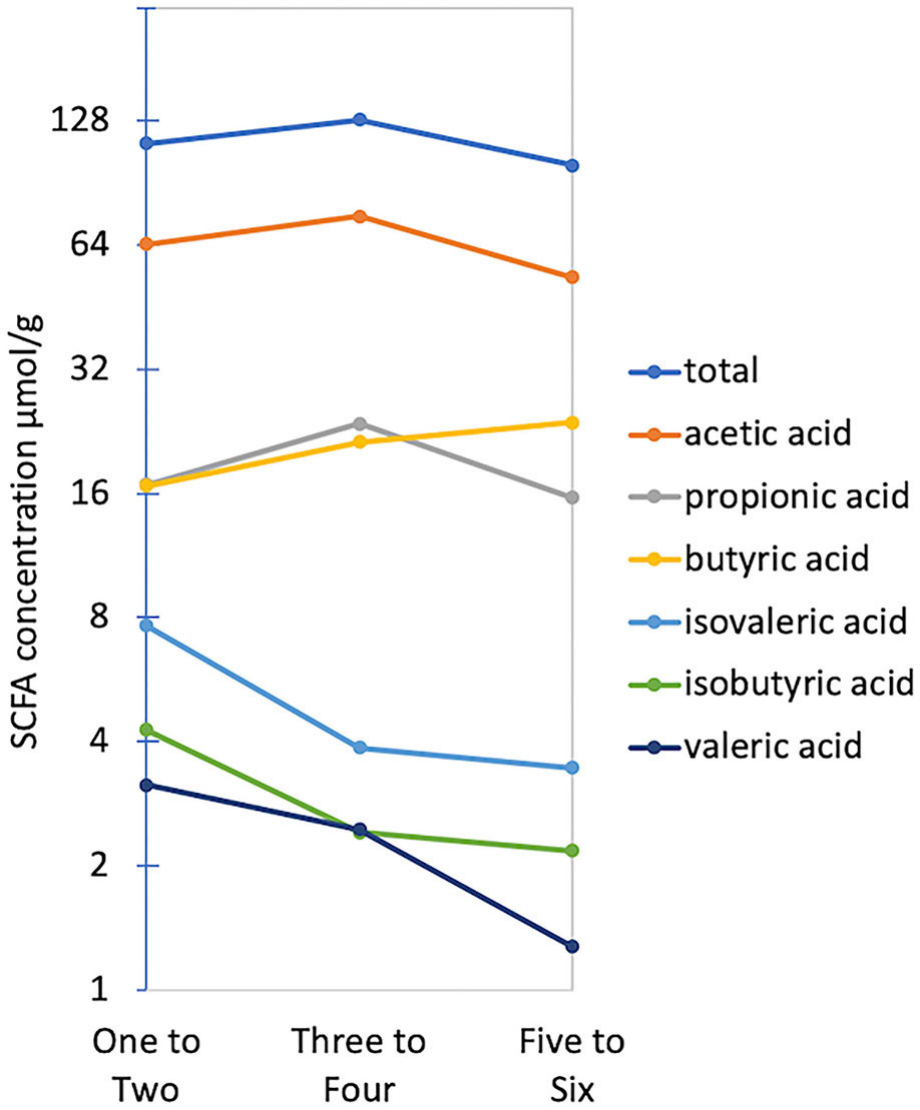
Average total and individual SCFA concentrations across Bristol stool form groups. The Y-axis is plotted on a log_2_ scale.

The average, total fecal SCFA concentration across all children was 119.01 μmol/g (95% CI 104.29–133.73) and the average molar ratio of acetate, propionate, and butyrate was 67:19:20. No significant differences in total or individual SCFA concentration were found between CSS (Figure 2C); however, children in the ASD group tended to have higher acetic acid, and total SCFA concentrations, and similarly, total and acetic acid concentrations negatively correlated with MSEL score (Figure 2E). The SCFA concentrations were also similar between the two treatment groups. When relating SCFA concentrations to stool form, the average concentration of acetate, propionate and the total SCFA concentration were all highest in normal stool samples, compared to a firm or loose stool samples, whereas iso-butyrate and iso-valerate were highest in firm stools, followed by normal and then loose stools. Butyric acid was the only acid that increased in concentration with stool looseness (Figure 5).

### Functional Analysis

**Figure 6:**
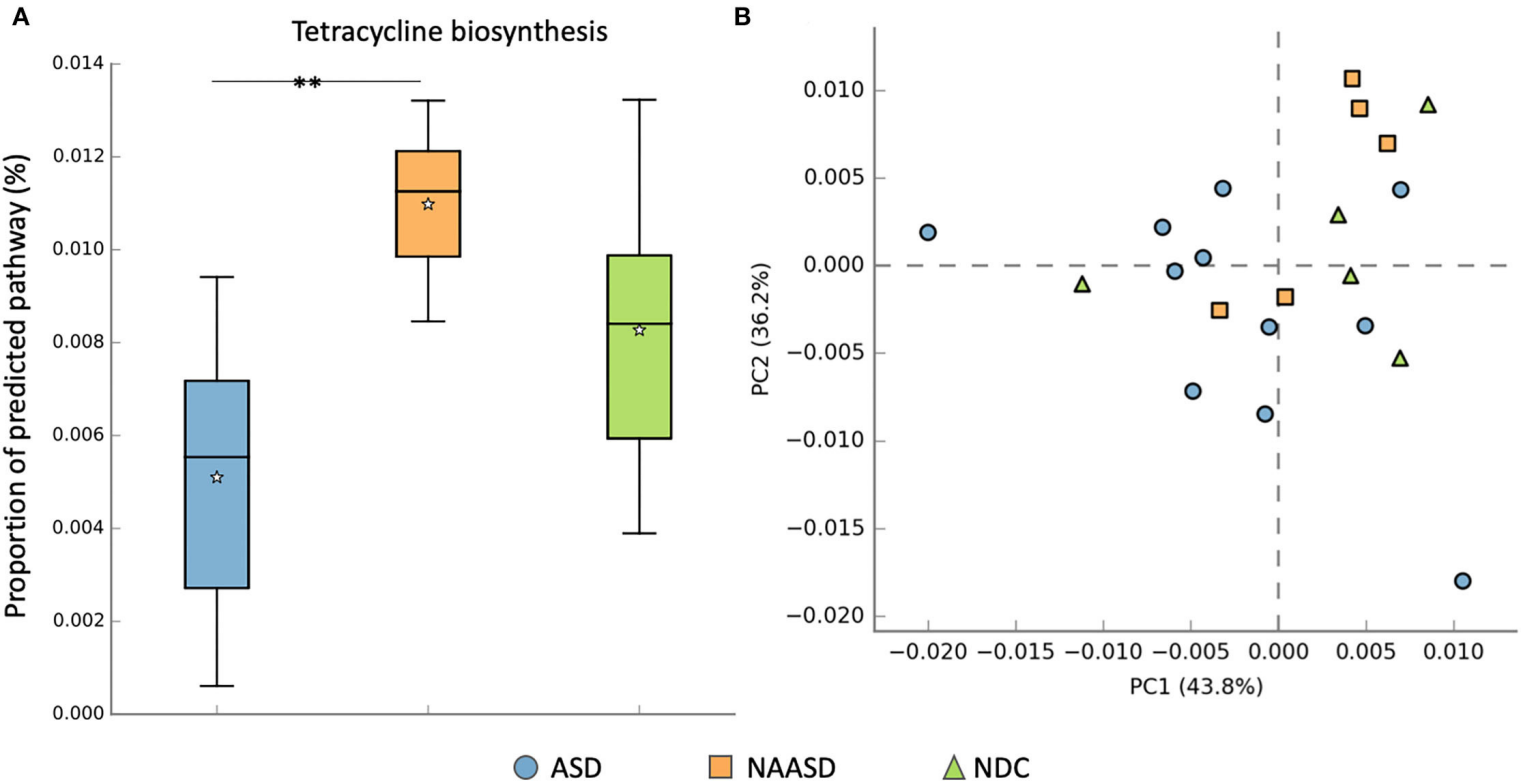
Differences in metabolic pathways between CSS groups. **(A)** Proportion of the predicted pathway tetracycline biosynthesis among CSS groups tested using Welch's *t*-test after FDR correction. **(B)** PCA distribution of predicted core metabolic pathways by CSS group. The * symbol indicates the significant difference after FDR correction *p* = 0.012.

Predictive community functional profiling using Tax4fun returned 321 functional pathways within Cellular Processes, Environmental Information Processing, Genetic Information Processing, human diseases, Metabolism, and Organismal Systems. On average 38.5% of ASVs and 52.7% of sequences per sample were mapped to a reference KO and used for prediction. After filtering to include only core metabolic functions, the profile consisted of 157 pathways within 13 classes. There was no visual clustering in the core metabolic profile between CSS groups using PCA (Figure 6), although a significant difference in the proportion of Tetracycline biosynthesis (after FDR correction), and several other pathways (before correction) was identified between CSS groups using Welch's *t*-test (Supplementary Table 5). Differences in the metabolic profile between intervention groups were also observed using PCA (Supplementary Figure 5), as well as individual metabolic pathways.

## Discussion

Identifying the behaviors associated with autism at a young age is possible, although predicting the trajectory of young children who are diagnosed with autism is typically not possible (Hyman et al., 2020). Roughly 80% of children who are diagnosed at <3 years of age will retain their diagnosis. However, because it is difficult to distinguish between either autism, or Pervasive Developmental Disorder-Not Otherwise Specified a conclusive diagnosis is difficult to make (Akshoomoff, 2006). Therefore, in this study, we used CSS and MSEL scores as indicators of autism symptom severity to explore the gut microbiome and SCFA associated with these young children. It is also important to note that the children in the no-developmental-concern group may not be considered neurotypical controls, as all children in this study were showing early behavioral signs of ASD determined by SACS-R at 9 – 14 months of age.

### Overall Composition of the Fecal Microbiome and SCFA Concentrations Among CSS Groups

Assessment of the bacteriome about autism has led to conflicting results, especially when comparing Bacteroidetes and Firmicutes (Iglesias–Vázquez et al., 2020); although ASD seems to be more often associated with a decrease in Firmicutes (Andreo-Martínez et al., 2021). In this study, there was no significant difference in the relative abundance of any phyla between children with ASD and NDC, and firmicutes were the most abundant and prevalent phyla, averaging 62% of the total proportion of reads across all children sampled. The abundance of Actinobacteriota dropped below 4% of the total community composition in the NAASD group, and a reduction in this phyla has also been observed previously in autistic children compared to neurotypical controls (Coretti et al., 2018). The NAASD group also had the highest levels of butyrate and propionate in the study and had a significantly elevated proportion of tetracycline biosynthesis based on predictive profiling. Tetracyclines are the most commonly used oral, broad-spectrum antibiotics, which can promote the proliferation of anaerobic bacteria (Kovtun et al., 2020).

Significant differences in both the community composition (Strati et al., 2017; Coretti et al., 2018; Pulikkan et al., 2018; Ma et al., 2019) and SCFA concentration (Adams et al., 2011; Wang et al., 2012; Coretti et al., 2018; Bojović et al., 2020) have also been observed between autistic and neurotypical controls. Changes in composition are likely to affect the functional microbiome and may help reveal the mechanisms, by which the microbial community may differ due to the ASD phenotype. SCFA concentrations in stool are good indicators of what was produced by resident bacteria as they reflect what was excreted after absorption by the host. Butyrate and propionate are elevated in stool samples from children with ASD compared to controls (Coretti et al., 2018). Considering children with a similar fiber intake, propionate has again been found in significantly higher concentrations in the stool of children with autism compared to neurotypical controls (Wang et al., 2012). This is particularly important because both acids can cross the BBB and can interact with brain cells *via* G-protein-coupled receptors (Abdelli et al., 2019). Although, in this study, we found no such difference in either beta diversity or SCFA concentration between CSS groups. Instead, butyrate was the only SCFA that increased with stool looseness. The similar levels of SCFA across CSS groups, but not Bristol stool groups, may indicate that stool form may be a confounding factor that makes it difficult to determine differences between neurological development, especially with small sample size. It may be that, at this young age, differences in the community structure associated with neurological disorders are less pronounced, but still present at a finer scale.

The average richness and diversity of the bacterial community also increased from the first (A) to second (B) time point in the six children that provided stool samples at both time points as did the number of bacterial species included in the core microbiome. As the diversity of a child's diet expands in early life, the microbiome also continues to develop and increase in diversity (Matamoros et al., 2013). Although, there was no significant difference in alpha diversity between CSS groups, and this is consistent with other studies with a larger number of young participants who were assessed for autism using various methods (Strati et al., 2017; Pulikkan et al., 2018; Kong et al., 2019; Fouquier et al., 2021). In this study, there was a trend for increased bacterial diversity within the NAASD group, as well as a trend toward a negative correlation between bacterial diversity and MSEL score. This negative correlation is in agreement with another study, which found three alpha diversity measures of the fecal microbiome at 1-year of age that were negatively correlated with both the Early learning Composite (a combination of 4 of the 5 standardized T-scores from the MSEL) and two of the five Mullen scales at 2-years of age (Carlson et al., 2018).

While these trends in alpha diversity may indicate new avenues for future investigation, we caution that it may be difficult to use traditional diversity indices to describe changes in the gut that are linked with less understood, heterogeneous, and modern diseases, such as ASD. Not to mention that the sequencing depth – the basis on which the diversity measures are calculated – varies considerably between studies, which may make it difficult to compare conclusions from different datasets (Willis, 2019). The inconsistency in alpha diversity about autism has also been mentioned in a review in this area (Krajmalnik-Brown et al., 2015). Here we propose that either an increase or decrease away from the diversity needed to maintain a healthy homeostatic microbiome may be disadvantageous. For example, an increase in diversity may indicate a bloom in pathobionts (Levy et al., 2017), or an increase in usually more transient bacteria, which may break down the normal community structure. Similarly, reduced diversity could indicate a loss of functionality of important microbial members and a community that is disordered (Levy et al., 2017).

### Species and Strain Differences in the Fecal Microbiome Between CSS Groups

To detect bacteria associated with ASD, we compared differential abundance between CSS groups. Using the BH adjustment to control for false positives, we did not detect any significant features, although, our investigation lacks statistical power using nonparametric tests with a small sample size, therefore, we discuss significant results prior to FDR correction. Overall members of Enterobacteriaceae and Negativicutes were increased only in the NDC group. Negativicutes are known for their contribution to propionate production (Reichardt et al., 2014) and *Veillonella* (Negativicutes) are known to ferment lactate to produce SCFA (Kang et al., 2013). *Veillonella* are reduced in the fecal microbiome of children with autism compared to neurotypical controls (Strati et al., 2017); whereas Enterobacteriaceae have been found to increase (De Angelis et al., 2013). Lachnospiraceae, including *Muricomes*, CHKCI001, and *Ruminococcus*, were also enriched in the NDC group compared to either the ASD or NAASD groups.

Taxa enriched in both non-neurotypical groups included members of Lachnospiraceae, including *Blautia* and an unresolved Lachnospiraceae species. *Blautia* is bile-metabolizing and associated with tryptophan metabolism, and has been found at the reduced abundance in a BTBR *T*^+^ *Itpr3*^*tf*^/J mouse model of ASD (Golubeva et al., 2017). This result is somewhat contradictory as *Blautia* are commonly associated with a typically developing infant microbiome (Hill et al., 2017), a healthy adult microbiome (Tap et al., 2009), and are well-known for their contribution to SCFA production (Louis and Flint, 2017). *Clostridium* p. was also more abundant in the ASD group, which is in line with other studies (Gondalia et al., 2012; Coretti et al., 2018; Ma et al., 2019). *Clostridium* is one group of bacteria known to be able to produce toxins that can cross the BBB (Góra et al., 2018), commonly found in both higher abundance (Iglesias–Vázquez et al., 2020), and numbers were based on CFU/g (Finegold et al., 2017) in fecal samples from children with autism compared to controls.

*Bacteroides caccae* has been associated with a decrease in abundance in the fecal microbiome of children with autism (Averina et al., 2020) but was enriched in the ASD group in this study. Certain strains of *B*. *caccae* are mucolytic, which may place them in close proximity to the host, where they can both influence and be influenced by the host (Tailford et al., 2015). Furthermore, myocytic bacteria may be opportunistic pathogens (Ganesh et al., 2013), and because complete degradation of mucins may require co-metabolism evolving several species (Tailford et al., 2015), shifts in an abundance of some members could impact the functionality of the total myocytic community, which, in turn, would be either beneficial or harmful to the host. While this finding adds to the catalog of autism-associated bacterial taxa, it may also indicate the importance of identifying different bacterial strains, as well as using complementary metabolite data to fully understand community changes between groups. Furthermore, the inconsistency seen in the literature regarding taxa associated with autism may be caused by differences in databases used for classification. This can be improved by a multi-omics study design, but also by collecting information that can influence microbiome composition, such as diet including fiber and protein content, as well as pre- or probiotic supplements, GI problems, and information on bowel movements.

### Stool Form May Help Explain Inconsistencies in the Bacteriome of Children With ASD

Poor stool form is an indication of GI distress, and issues, such as constipation, diarrhea, and flatulence, are reported as more prevalent in children with ASD than in children with neurotypical development (Chaidez et al., 2015). The way GI symptoms are documented could result in variation; for example, if study participants assess their stool using the Bristol Stool Scale, there may be individual bias, or error if recalling stool form after the collection has happened. In this study, stool form was assessed during sample processing, which allowed for a non-bias assessment. We found differences in bacterial composition between firmer stools compared to normal or loose stools. This finding is in agreement with another study comparing autistic and neurotypical controls (including sibling controls), where stool consistency was among the factors most strongly associated with the microbiome composition (Yap et al., 2021). However, Yap and colleagues proposed a top-down impact on stool composition beginning with reduced dietary diversity due to self-restricting eating habits, and subsequently reduced microbiome diversity resulting in loose stool consistency.

In our study, both richness and diversity were lower in firmer stool samples compared to normal or loose stool (data not shown). Stool becomes firm with lower water content, and has been linked to longer whole gut transit times (Saad et al., 2010), which may impose selective pressures on the microbial community, particularly if the firm stool is experienced frequently. This same trend in reduced microbial diversity in firm stool has been observed in adults with functional constipation (Huang et al., 2018). In our study SCFA concentrations (except for butyric acid) were also reduced in looser stools, which is likely due to both water content, and the potential for microbial members to influence and be influenced by gut motility (Zhao and Yu, 2016). Therefore, our results are in line with the emerging hypothesis that the microbiome is part of a circular feedback loop, where behavior and environment have a top-down effect on the microbiome and stool form, and the microbiome imposes a bottom-up effect. Collecting GI disturbance metadata, as well as recording stool form (as this may change even within a day) and using this information as a potential confounding variable might help explain some of the observed inconsistencies in ASD-associated bacteria.

### Limitations

The number of children in this study was limited and the number of children in diagnostic and treatment groups was small and unbalanced, and no information regarding diet and gastrointestinal distress was collected. Also, because the children were first recruited prior to diagnosis, it was not possible to choose the number of children in each category. As a result, the number of children with no developmental concerns were quite small. Additionally, the diagnosis of the children in the iBASIS-VIPP and UCC groups were mixed, and due to the sample size, it was not possible to examine differences between diagnoses within the intervention group. Using full siblings residing in the same household as the healthy control group is the most reliable control group as it covers both environmental factors and genetic background; however, this would also not be possible with this pre-emptive study design. We would also like to point out that there are numerous methods used to diagnose autism severity, and, therefore, within the literature we discuss several diagnostic tools have been used. Lastly, due to the small sample size, we chose to display results that were significant prior to correction for multiple testing. While this less stringent approach may lead to more false positives, it may also indicate those taxa that could be important in the shifts taking place in these young children that otherwise would be missed.

### Conclusion and Future Research

In this study, the relative abundance of the microbiome at the phylum level, and the diversity of bacterial and fungal communities were similar when viewed among neurological developmental groups. The microbiome composition and SCFA concentrations were, instead, found to be significantly associated with stool form, indicating that this factor might be important to consider when interpreting the microbiome composition of young children with autism – especially considering the high prevalence of gastrointestinal issues in children with ASD. While bacterial or fungal diversity could not be used to discriminate between neurological development, differential abundance in the community structure at the genus, species, and strain level was detected between CSS groups. Together, these findings indicate that subtle changes in the bacterial composition may occur in the microbiome of young children with autism. We also found that the amount of inter-individual difference in the microbiome from the 6 children over two time points, did not seem to be consistent, or related to autism severity. This is likely due to the small number of samples, but it would be beneficial to collect a longitudinal series of samples along with diet and bowel movement history to establish the influence of these factors on the development of young children showing early behavioral signs of ASD.

Lastly, pre-emptive treatment for children at risk of developing autism is an important research area as there is currently no cure for autism, and treatments that begin before diagnosis – earlier in life – may be more effective than those started later in life. The children in this study were involved in a broader study examining the efficacy of behavioral interventions for children showing early signs of ASD (Whitehouse et al., 2021). Although neither behavioral intervention arm involved dietary changes, and the sample size was very small, significant differences in bacterial community structure were observed between the intervention groups. While it is unclear how the behavioral intervention could impact the structure of the microbiome, the differences observed at multiple taxonomic levels between the two treatment groups may indicate an effect on the microbiome that warrants future research addressing the possibility of a top-down effect of cognitive or behavioral changes, which selectively benefit some bacterial groups. However, this potential needs to be investigated in a much larger study to account for the many factors affecting the gut microbiome in the developing child.

## Data Availability Statement

The datasets presented in this study can be found in online repositories. The name of the repository and accession number can be found below: Figshare, https://figshare.com/s/fb83fed3102d7d437920.

## Ethics Statement

The studies involving human participants were reviewed and approved by Human Research Ethics Committee, Curtin University, Western Australia. Written informed consent to participate in this study was provided by the participants' legal guardian/next of kin.

## Author Contributions

CC and JJ conceived the study design. JJ conducted all laboratory work and wrote the manuscript. JJ, CC, SR, and MM-D analyzed the data. All authors revised the manuscript. All authors contributed to the article and approved the submitted version.

## Conflict of Interest

The authors declare that the research was conducted in the absence of any commercial or financial relationships that could be construed as a potential conflict of interest.

## Publisher's Note

All claims expressed in this article are solely those of the authors and do not necessarily represent those of their affiliated organizations, or those of the publisher, the editors and the reviewers. Any product that may be evaluated in this article, or claim that may be made by its manufacturer, is not guaranteed or endorsed by the publisher.
