# Supplementary material for: Changes to the Gut Microbiome in Young Children Showing Early Behavioral Signs of Autism
Source: Front Microbiol. 2022 Jul 28;13:905901. doi: 10.3389/fmicb.2022.905901 (PMC9371947; doi:10.3389/fmicb.2022.905901)
Supplement: Supplementary file 1 [file Data_Sheet_1.docx]

Supplementary Material

# Supplementary Figures and Tables

## Supplementary Figures


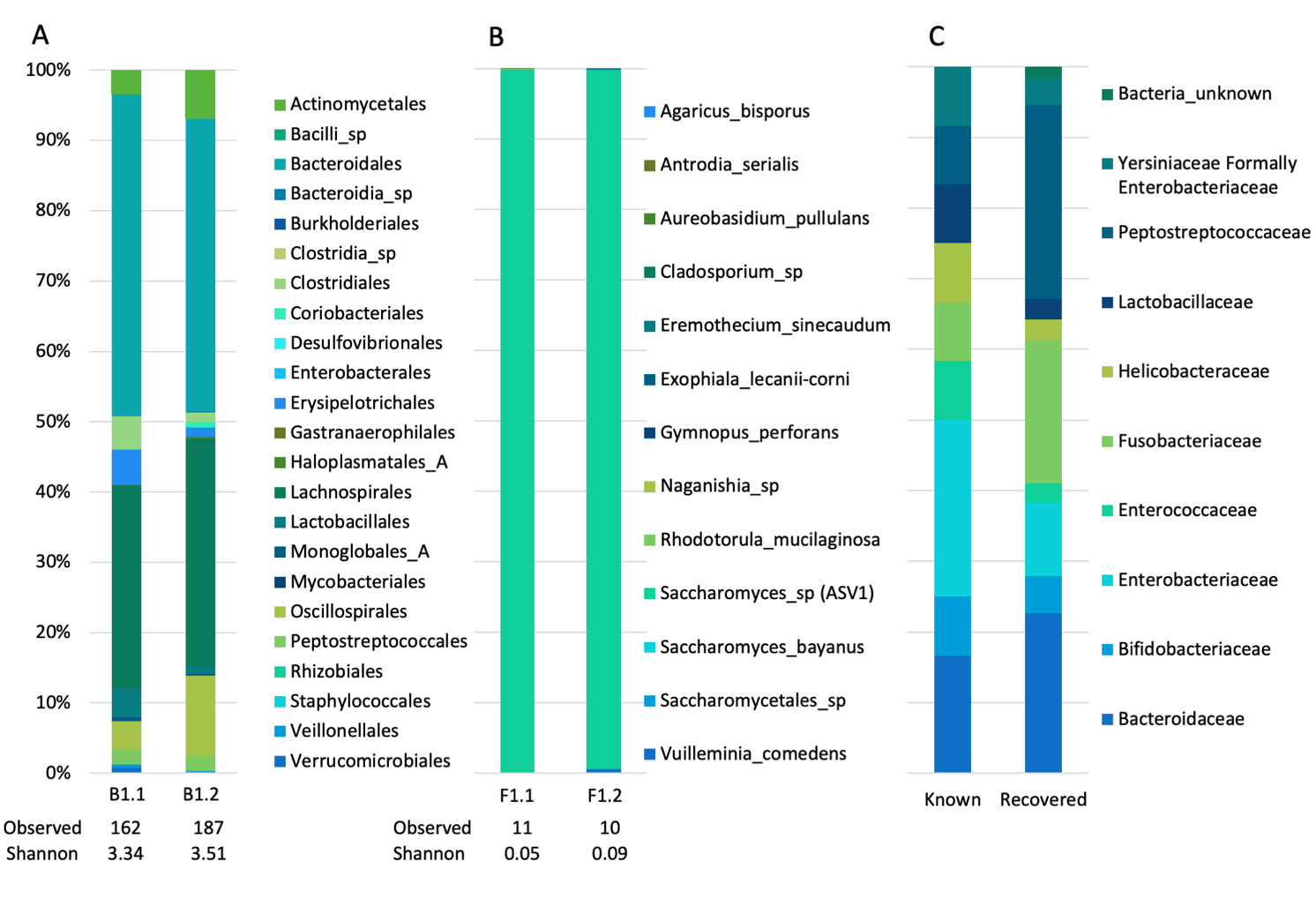


**Supplementary Figure 1.** Replicate sampling of a single individual, showing the Bacteriome at the level of Order (A), and mycobiome at the level of ASV, with species assignments shown (B). The mycobiome replicate samples include ASV 1, which dominated both replicates F1.1 and F1.2 with 165,517, and 105,517 reads per sample respectively. Richness and Diversity were calculated on filtered non-rarefied counts. A Bacterial mock community (C) shows the proportion of both the known composition of the mock community positive control at the family level, and the recovered mock community sample composition.

**
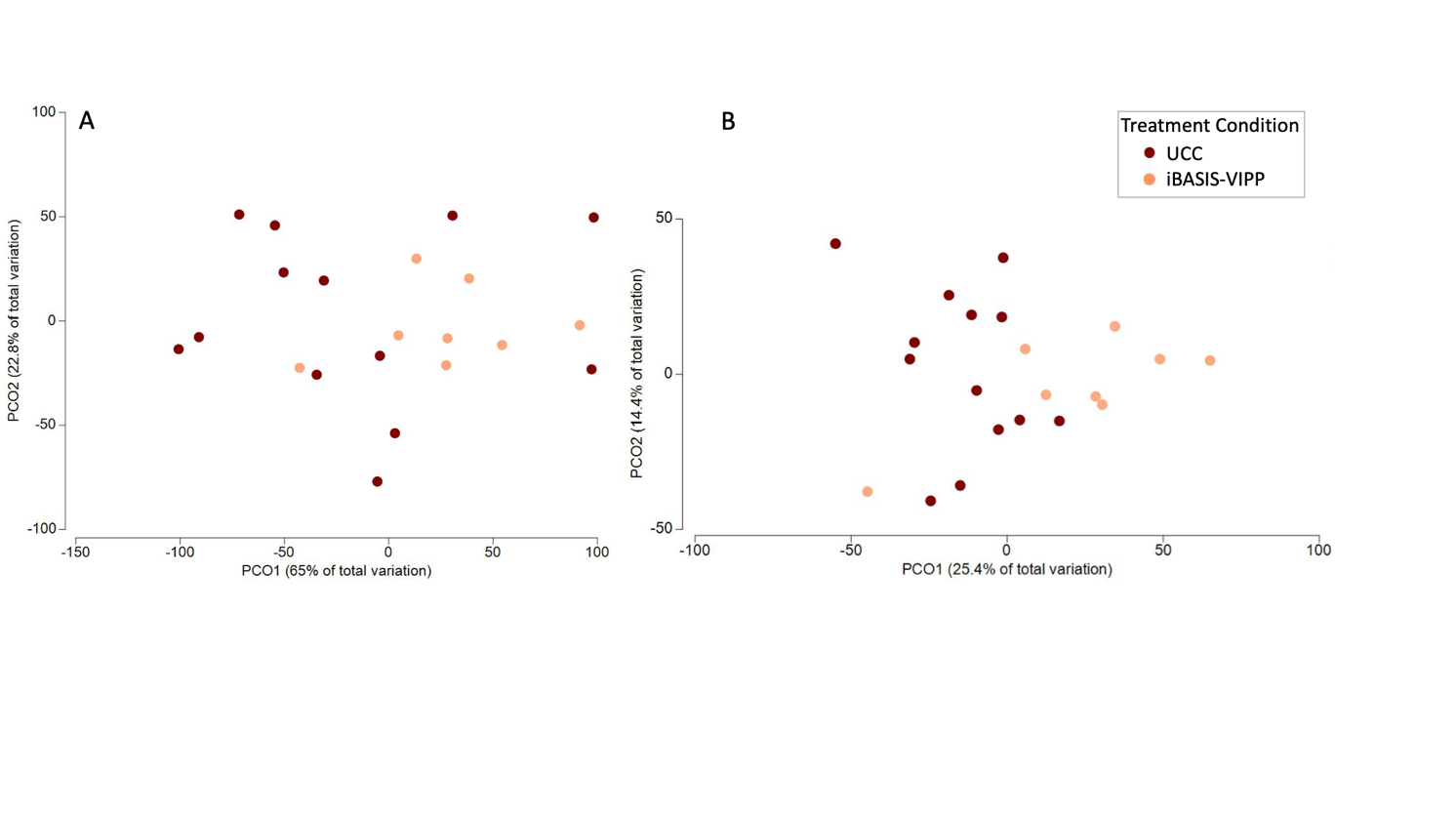
**

Supplementary Figure 2. PCoA plots showing clustering of bacterial communities observed by treatment in the AICES study, at the level of phyla (A) and genus (B). Differences were calculated on Euclidian distance of CLR transformed counts.


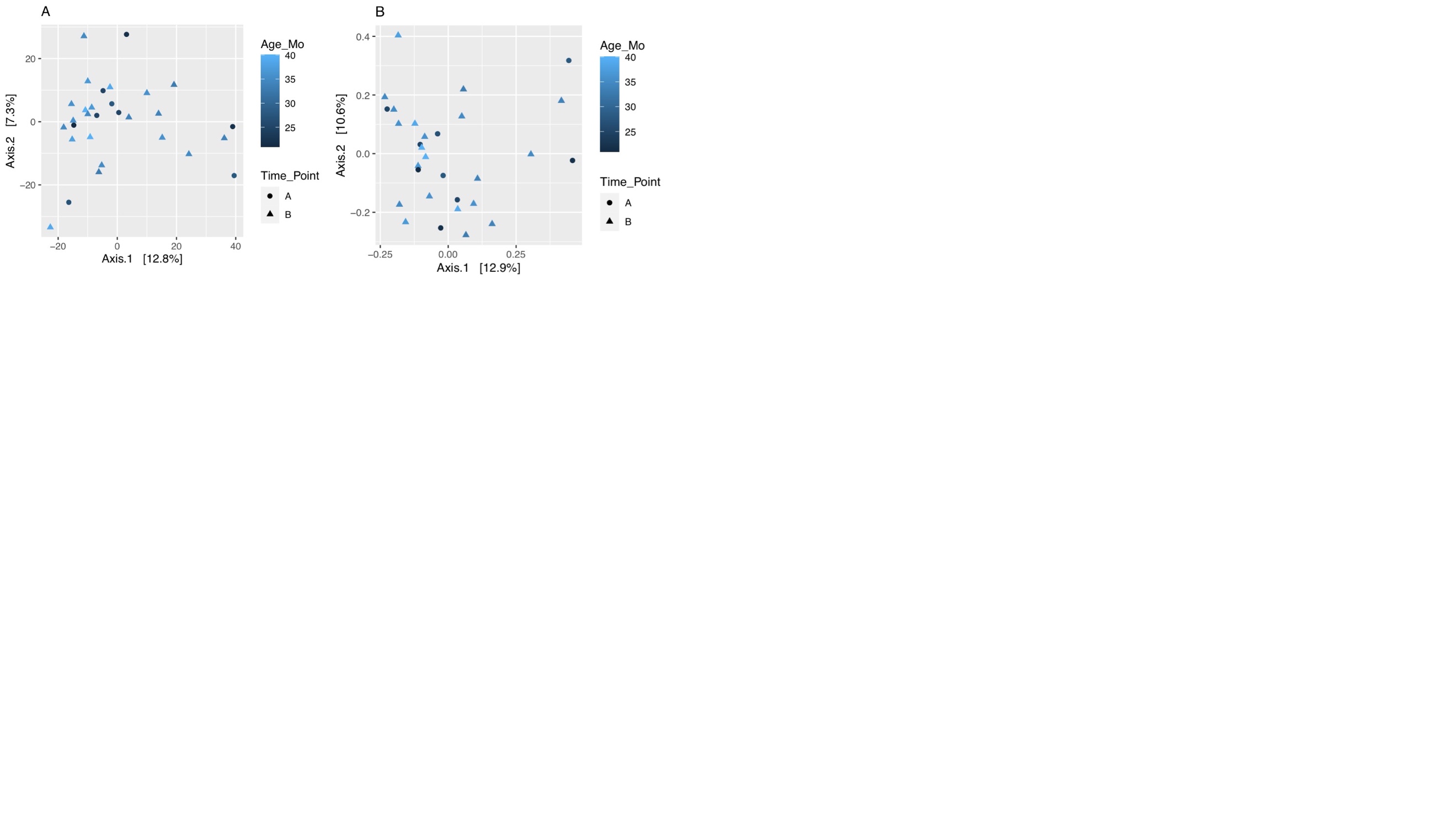


Supplementary Figure 3. Clustering of bacteriome samples according to PCoA using Euclidian distances of CLR transformed counts. Samples are identified by age in months, and the sampling timepoint.

Supplementary Figure 4. Four bacterial families with differential abundance (CLR) according to stool form. The number of children in each stool form group was 4, 12, and 5 in the one-two, three-four, and five-six groups respectively.


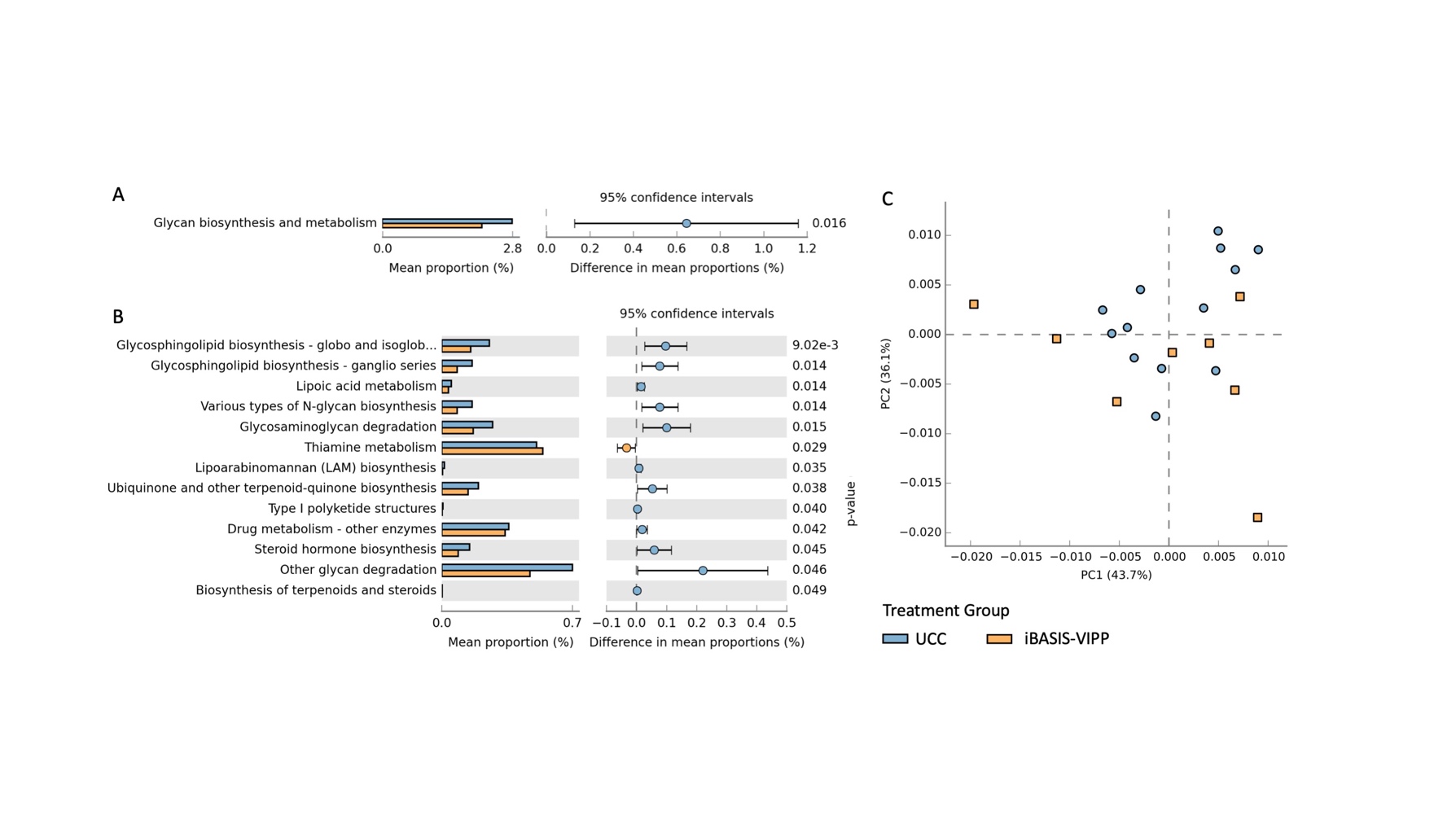


Supplementary Figure 5. Differences in predicted pathway class (A), and individual pathways (B) associated with treatment type, and clustering via PCA of individual pathways based on treatment group (C). All plots were based on the filtered core metabolic predicted pathways.

## Supplementary Tables

Supplementary Table 1. Distribution of reads in each microbial dataset after quality filtering, and in the dominant microbiome subset

Supplementary Table 2. Taxa observed with differential abundance between preemptive treatment groups. Significant differences were determined using the Mann-Whitney U method using only dominant bacterial taxa.

Supplementary Table 3. Genera identified as having differential abundance between lose (Bristol Stool number 1-2), normal (Bristol Stool number 3-4), and firm (Bristol Stool number 5-6) stool. P values and FDR corrected values are shown after Kruskal-Wallis test.

Supplementary Table 4. Bacterial and Fungal taxa with differential abundance between CSS groups. Only dominant bacterial and fungal taxa were included.

Supplementary Table 5. Individual pathways which differ significantly prior to FDR correction between CSS groups using Welch’s t-test.

# Supplementary results

Bacterial data resulted from all 30 fecal samples, with a minimum of 55,418 reads per sample, and 1,165 ASVs from eight phyla. The Fungal data resulted from 29 samples and a minimum of 1095 reads per sample with 231 ASVs from two phyla prior to prevalence filtering (Sup Table 1). A single sample was also processed in duplicate to visualize the bias due to library preparation for both bacteria and fungi (Sup Figure 1). At the level of order, bacteriome communities showed high similarity, with the read count of Oscillospirales, most variable between the replicate samples (B1.1 = 4%; B1.2 = 11.5%). The mycobiome was compared at the level of ASV (species IDs shown) because of the lower diversity of this community. The total read count was much higher in the fungal samples >100,000 reads, and the communities were both dominated by ASV1, with a read counts 100x higher than all other ASVs. The mycobiome was also more variable with 57,587 more reads in F1.1 compared to F1.2 verses the bacterial replicates where B1.1 had 2,251 more reads than B1.2, which is typical of sequencing technology which generates stochastic variation in sequence counts. The American Type Culture Collection (ACTT) bacterial mock community (https://www.atcc.org/products/msa-1006), was also sequenced as a positive control (Sup Figure 1), containing 12 species in even concentrations from 9 families including multiple species in Bacteroidaceae, and Enterobacteriaceae. With greater than 500 reads, 17 ASVs were recovered from the sample mock community, from the correct nine families. Twelve ASVs were assigned to the correct 12 genera, and 11 ASVs were assigned to the correct species at 100% identify; with 7 of these ASVs matching to two or more
